# Development and characterization of microsatellite loci for the endemic *Thalictrum smithii* (Ranunculaceae)

**DOI:** 10.1002/aps3.1176

**Published:** 2018-08-22

**Authors:** Bin‐Bin Du, Ya Zhan, Jiao‐Kun Li, Ying‐Zhuo Chen, Lu‐Lu Tang

**Affiliations:** ^1^ School of Life Sciences Central South University Changsha 410013 People's Republic of China; ^2^ School of Minerals Processing and Bioengineering Central South University Changsha 410083 People's Republic of China; ^3^ Chengnan Academy Hunan First Normal University Changsha 410205 People's Republic of China

**Keywords:** genetic diversity, microsatellites, polymorphism, Ranunculaceae, *Thalictrum*, *Thalictrum smithii*

## Abstract

**Premise of the Study:**

Microsatellite primers were developed for the gynodioecious Chinese endemic *Thalictrum smithii* (Ranunculaceae).

**Methods and Results:**

Thirty‐nine microsatellite primers were developed using the Fast Isolation by AFLP of Sequences COntaining repeats (FIASCO) method. Thirteen microsatellite loci were found to be highly polymorphic after screening 114 specimens (60 hermaphrodite and 54 female) from three *T. smithii* populations. The number of alleles per locus ranged from three to 13, and the levels of observed and expected heterozygosity per locus ranged from 0.000 to 1.000 and from 0.204 to 0.834, respectively. Twenty‐six of these primers were polymorphic in *T. petaloideum* and *T. finetii*.

**Conclusions:**

These markers will be useful for examining genetic diversity, polyploidy, and mating system in populations of *T. smithii* and for guiding study on the evolution of speciation in *Thalictrum*.

Evolutionary transitions of sexual systems from hermaphrodite to unisexual have repeatedly occurred in different lineages of flowering plants. Transitions between sexual systems are often associated with changes in pollination modes. For example, many wind‐pollinated species have unisexual flowers (Delph et al., 2007; Friedman and Barrett, 2008). The genus *Thalictrum* L. (meadow‐rues; Ranunculaceae) has been considered an ideal group to explore the evolutionary transitions in sexual systems and pollination modes. There are numerous hermaphrodite and dioecious species, which are insect‐ or wind‐pollinated (Boivin, 1944; Soza et al., 2012, 2013). The evolutionary transition from hermaphrodite to dioecy has been repeatedly observed in diverse lineages of flowering plants via an intermediate stage of gynodioecy, a dimorphic sexual system in which female and hermaphrodite individuals co‐exist within the populations (e.g., Delph et al., 2007). Of the approximately 70 *Thalictrum* species found in China, our recent investigations in the Hengduan Mountains revealed that *T. smithii* B. Boivin was the only known gynodioecious species in this genus.

Microsatellite or simple sequence repeat (SSR) markers are co‐dominant with high levels of polymorphism and have been widely used in studies of genetic diversity, genome mapping, mating systems, and genetic differentiation (Jarne and Lagoda, 1996; Zhang and Hewitt, 2003; Li et al., 2015a). We developed and characterized 13 polymorphic microsatellite loci for *T. smithii* and tested the applicability of these SSR loci in three wild populations with different sex ratios to estimate genetic diversity of *T. smithii*.

## Methods and results

Three *T. smithii* populations were sampled in western Sichuan Province, China (Songgangcun [SGC], Xianggelilazhen [XGLL], and Yajiangxian [YJX]; Appendix 1). Fresh leaves were collected from up to 20 randomly chosen individuals per sex type from each population; individuals were at least 10 m apart from each other. Furthermore, two monoecious *Thalictrum* species were collected in the Hengduan Mountains: *T. petaloideum* L. and *T. finetii* B. Boivin. Location, sample sizes, and sex ratio of these sampled populations are listed in Appendix 1. All flowering individuals were counted in each population to estimate the sex ratio.

Microsatellite loci were developed according to the method described by Li et al. (2015b). Using the QIAGEN DNA Extraction Kit (QIAGEN, Hilden, Germany) and the cetyltrimethylammonium bromide (CTAB) method (Porebski et al., 1997), total genomic DNA was extracted from the leaves of *T. smithii* sampled from the SGC population.

The Fast Isolation by AFLP of Sequences COntaining repeats (FIASCO) procedure was used for isolating microsatellite loci from an enriched (AG)_n_ library (Zane et al., 2002). After being digested with *Mse*I (Fermentas, Burlington, Ontario, Canada), genomic DNA was ligated to an *Mse*I‐adapter pair (F: 5′‐TACTCAGGACTCAT‐3′, R: 5′‐GACGATGAGTCCTGAG‐3′) with T4 ligase (Promega Corporation, Madison, Wisconsin, USA). The diluted (1 : 10) digestion‐ligation mixture was directly amplified with *Mse*I‐N primers (5′‐GATGAGTCCTGAGTAAN‐3′; 25 μM) in a total volume of 20 μL using the following thermocycler conditions: initial denaturation at 95°C for 3 min; followed by 26 cycles at 94°C for 30 s, annealing at 53°C for 1 min, and extension at 72°C for 1 min; and a final extension at 72°C for 5 min.

Microsatellite repeats were selected using magnetic beads with 5′‐biotinylated (AC)_15_ and (AG)_15_ probes, which enriched amplified DNA fragments in the range of 200–800 bp. Nonspecific DNA fragments were removed by three non‐stringency washes with TEN1000 (10 mM Tris‐HCl, 1 mM EDTA, 1 M NaCl [pH 7.5]) followed by three stringency washes using 0.2× saline sodium citrate (SSC) and 0.1% sodium dodecyl sulfate (SDS). The enriched DNA fragments were then amplified via PCR using the primers *Mse*I‐N for 26 cycles. After purification with a Gel Extraction Kit (TaKaRa Biotechnology Co., Dalian, Liaoning, China), the PCR products were cloned into the pGEM‐T vector (Promega Corporation) and transformed into DH5α cells (TransGen Biotech, Beijing, China). Recombinant clones were screened by blue/white selection, and the positive clones were tested by PCR using primers T7 and Sp6 separately to screen (AC)_10_‐rich or (AG)_10_‐rich clones.

The total of 96 clones with positive inserts were sequenced on an ABI 3730xl Genetic Analyzer (Applied Biosystems, Foster City, California, USA). Using the software SSR Hunter (Li and Wang, 2005), microsatellite repeat motif regions were identified. Among the 96 sequences analyzed, 77 contained microsatellite motifs. Thirty‐nine high‐quality sequences were selected after exclusion of redundant sequences, and the software OLIGO 7.0 (Rychlik, 2007) was used to design microsatellite primers with the following criteria: product length 80–300 bp, primer length 21 bp, melting temperature 48–68°C, GC content 27–60%. Fluorescent dyes 6‐FAM, TAMRA, or HEX (Invitrogen, Carlsbad, California, USA) were used to label the 39 pairs of primers (Table 1). Among three *T. smithii* populations (Appendix 1), 114 individuals were randomly selected to assess polymorphism and performance for the 39 SSR loci. Amplifications were performed in a 25‐μL reaction system containing 30–50 ng of genomic DNA, 0.6 μM of each primer, and 7.5 μL of 2× *Taq* PCR MasterMix (0.1 unit *Taq* polymerase/μL, 0.5 mM dNTP each, 20 mM Tris‐HCl [pH 8.3], 100 mM KCl, 3 mM MgCl_2_; Tiangen, Beijing, China). The thermocycling conditions were: 94°C for 5 min; 32 cycles at 94°C for 30 s, at an annealing temperature optimized for each specific primer for 30 s (Table 1), and at 72°C for 60 s; and a final extension at 72°C for 5 min. The PCR products were separated using an ABI PRISM 3730xl DNA sequencer with GeneScan 500 LIZ Size Standard (Applied Biosystems) and were genotyped using GeneMapper version 4.0 (Applied Biosystems).

**Table 1:** Characteristics of 39 microsatellite loci developed for *Thalictrum smithii*

Locus	Primer sequences (5′–3′)	Repeat motif	Allele size range (bp)	*T*_a_ (°C)	Fluorescent dye	GenBank accession no.
Tsp01	F: CAGGTATATTGCTTCCTTTAC	(AG)_10_	100–140	56	6‐FAM	KY243242
	R: ATGGCTGGCCTCCTTGTTTGG					
Tsp02	F: ATCAATGGCTATGAACATCTA	(AG)_14_	172	53	HEX	KY243241
	R: CATCCTAAACACCTATCTCTT					
Tsp03	F: TTGAGAAAAACAGAGATGAAA	(AG)_4_(TG)_5_(AG)_9_	145–170	54	HEX	KY243240
	R: CAATAGCCTAAAACGGACACC					
Tsp04	F: GATGGAAAAAATTCGAGCAGA	(CT)_11_	92–122	54	6‐FAM	KY243239
	R: ATTCGGGAGAGGAGAGCAAAA					
Tsp05	F: AAAACGAAATTACACCAACCG	(TC)_16_	123–158	51	6‐FAM	KY243238
	R: TGCCCTGAAAGAGAACCACAT					
Tsp06	F: CCCAAGATAGTTTACTCCGAG	(CT)_12_	125–137	53	6‐FAM	KY243257
	R: CAGTTGAAAGTGTTGACCTGA					
Tsp07	F: AAGAGAATAGCGTGAGAGATA	(AG)_8_	185–240	51	HEX	KY243256
	R: AAAACAGAGAAGAAACAAAAA					
Tsp08	F: GCTGCACATCACTACTCACT	(GA)_13_	279–295	50	6‐FAM	KY243255
	R: CAACACTTCATTCCCTTTTT					
Tsp09	F: AACAGATAATTCTCACCTTTG	(AG)_13_	92–116	56	HEX	KY243254
	R: CCTTACACCTTTTGCTCCCGC					
Tsp10	F: ATTCCCGATTCCAACTATAAA	(TC)_9_	98–125	55	6‐FAM	KY243253
	R: CGATCAAGAATTTGGGATTTT					
Tsp11	F: AATGTAGCAAGCTATGGTACA	(AG)_10_	122–132	54	6‐FAM	KY243252
	R: CTCCACTAAATGCCTTTCAGA					
Tsp12	F: CACTCTTGTCACTCACTCTCT	(GA)_9_	230–270	52	HEX	KY243251
	R: TTACTAAACCCAAATCATCTA					
Tsp13	F: TTAATCAAATTGAAACCTAGA	(AG)_12_	179–201	50	6‐FAM	KY243250
	R: CAAGAAAAGAAAGAAGAGAAT					
Tsp14	F: GCCTTGTTCAACCCTCCATAC	(CT)_10_	100–110	51	HEX	KY243249
	R: TACTACTTCTACCTTTCTTTC					
Tsp15	F: TCACACCTCTTCCACCACATT	(TC)_10_	92–108	50	TAMRA	KY243248
	R: AATTCTGGTTTTCTGGCCGGA					
Tsp16	F: TTTTGATTTTCTCTCTCTTCTA	(CT)_11_	116–140	52	HEX	KY243247
	R: CTTGAGATAGGTTAGTGTACCA					
Tsp17	F: TCCTACAGCCAGCGAATCAATG	(TC)_11_	128–164	50	6‐FAM	KY243246
	R: ACACCACTTCGTCAGACGGTCA					
Tsp18	F: TCTACTCTGTTCTTCTGCTGG	(TC)_12_	203–241	52	HEX	KY243245
	R: TAAAAAATGGGGTACCTTGCG					
Tsp19	F: CGGAAGGAGATTGAAGAAGGA	(CT)_10_	212–216	58	TAMRA	KY243244
	R: TGCCCAAGGGGGTTGGGTATG					
Tsp20	F: ACTTCTCTCTTTCTCTTCTCC	(CT)_12_	115–150	54	6‐FAM	KY243243
	R: GATTGATGCTTTTTTTTTATT					
Tsp21	F: ATTCAAAGCCTGTGTCAGATC	(AG)_10_	120–140	55	6‐FAM	KY243265
	R: GCAGCCATTCTTATTTCATTT					
Tsp22	F: TATCTTTCATTCACTCCACAC	(TC)_9_	86–105	58	6‐FAM	KY243264
	R: ACCTAATTAGCCGCATGAAGC					
Tsp23	F: TTAAGAACAGACATCGATCCA	(TC)_9_	120–132	56	6‐FAM	KY243263
	R: TCAGAAACAGTAAACACAAAG					
Tsp24	F: TTAAGGAAAACCGAGAAGCAT	(AG)_9_	96	52	TAMRA	KY243262
	R: TTCCAAGATGAAACGAGTAAA					
Tsp25	F: CACCAGTAACAGAAACAAAAA	(GT)_8_	160–180	50	6‐FAM	KY243261
	R: GCCACAAGTCAATTCCAAGAT					
Tsp27	F: ATCTCAGTTTCAGTACTTTCT	(CA)_8_	123–138	52	6‐FAM	KY243259
	R: TATATTCTCTTTCAATTTTTC					
Tsp28	F: ATCAGAACTGTCTCAGGCTTT	(CA)_7_	140–158	54	6‐FAM	KY243258
	R: TAATTGGTTGGCATTATGTCT					
Tsp29	F: GTTATGGCTAGAATGTTTTGTT	(TA)_8_	142–162	55	6‐FAM	KY243277
	R: TGTTGGTCTCAGCTTGAGAGGG					
Tsp30	F: GAATTCGATATAGACTACTTC	(CA)_9_	118–128	50	6‐FAM	KY243276
	R: AATAATGTAGACAATCTAGTC					
Tsp31	F: TCATTCTTCTGCTTCCTTTGC	(TG)_9_	169–202	56	HEX	KY243275
	R: CCACTACCTCCATCATCACCT					
Tsp32	F: ACTTTACTACCACTTCCTCAA	(GT)_9_	200–224	52	6‐FAM	KY243274
	R: AGCCACAAACATGCCACCTTC					
Tsp33	F: AAGGTAGCTGCTACAAGATCA	(AC)_6_	100–120	54	6‐FAM	KY243273
	R: TTCTGAGTACCAACCACTTCA					
Tsp34	F: ATTGATGTTTTGATTTTTTGG	(CA)_7_	105–122	50	6‐FAM	KY243272
	R: AATTTAGCTGGATTTGCTTAG					
Tsp35	F: GCGACTGTCAAACTTTAGAGA	(TG)_8_	80–110	52	6‐FAM	KY243271
	R: TTAGCGGCAAATGTAATTATC					
Tsp36	F: TGCCTTTTATGGTCAGATTCC	(TG)_6_	200–330	53	HEX	KY243270
	R: TATTTTTGTTGCTCACGTTGG					
Tsp37	F: TCACCAAAATAATAACGGCAC	(AC)_9_	97–121	53	6‐FAM	KY243269
	R: ATCCAAAATGGACACCAGACC					
Tsp38	F: TTGTCACAAGTTTACAAGACA	(CA)_8_	183–200	52	TAMRA	KY243268
	R: GAAGCATAGGTAGAGGAAGGA					
Tsp39	F: ACCTGGAATGTTCAGAAAGAT	(CT)_6_NNN(AC)_11_	128–164	52	6‐FAM	KY243267
	R: AACGACTTAGAAAATGGCAGA					
Tsp40	F: AATTTACAGTACATACTTGGC	(AC)_7_	125–160	51	TAMRA	KY243266
	R: TTAACTCTAGATAGGTCTTGG					

Note

*T*_a_ = annealing temperature.

Of the 39 primer pairs, 13 displayed polymorphism among individuals from the three *T. smithii* populations (Table 2). CERVUS version 3.0.7 (Kalinowski et al., 2007) was used to analyze the number of alleles per locus (*A*), observed and expected heterozygosity (*H*_o_ and *H*_e_), and deviation from Hardy–Weinberg equilibrium. Across the three *T. smithii* populations, *A* ranged from three to 13, with a mean of 5.308 in the SGC population, 6.308 in the XGLL population, and 6.846 in the YJX population. Levels of *H*_o_ and *H*_e_ per locus ranged from 0.000 to 1.000 and from 0.204 to 0.834, respectively. A relatively high level of genetic diversity was found in the SGC population (*H*_o_ = 0.489, *A* = 5.308) compared with the XGLL population (*H*_o_ = 0.346, *A* = 6.308) and the YJX population (*H*_o_ = 0.437, *A* = 6.846). Some loci showed significant deviation from Hardy–Weinberg equilibrium (Table 2).

**Table 2:** Results of initial primer screening of 13 polymorphic microsatellite loci in three populations of *Thalictrum smithii*.^a^

Locus	SGC (*N* = 34)			XGLL (*N* = 40)			YJX (*N* = 40)		
	*A*	*H*_o_	*H*_e_ b	*A*	*H*_o_	*H*_e_ b	*A*	*H*_o_	*H*_e_ b
Tsp04	9	0.618	0.722	7	0.450	0.597	8	0.450	0.768**
Tsp08	6	0.500	0.702	3	0.075	0.420	9	0.750	0.834
Tsp09	4	0.853	0.617*	6	0.625	0.516	5	0.750	0.602
Tsp13	4	0.382	0.673**	7	0.125	0.774**	8	0.325	0.802
Tsp15	5	0.235	0.445	3	0.025	0.204	5	0.200	0.640**
Tsp16	4	0.382	0.559*	4	0.150	0.670**	5	0.300	0.692**
Tsp17	4	0.500	0.712**	11	0.450	0.769**	9	0.675	0.829
Tsp18	6	0.794	0.767	12	0.500	0.803**	13	0.700	0.791
Tsp19	3	0.000	0.381	3	0.000	0.466**	3	0.050	0.507**
Tsp31	5	0.235	0.495**	6	0.300	0.574**	7	0.225	0.708**
Tsp32	5	1.000	0.569**	4	0.875	0.664**	7	0.575	0.492
Tsp37	9	0.500	0.757**	6	0.525	0.628	7	0.625	0.781
Tsp39	5	0.353	0.614**	10	0.400	0.828**	3	0.050	0.224
Average	5.308	0.489	0.616	6.308	0.346	0.609	6.846	0.437	0.667

Note

*A* = number of alleles; *H*_e_ = expected heterozygosity; *H*_o_ = observed heterozygosity; *N* = sampled individuals from each population.

^a^See Appendix 1 for locality and voucher information.

^b^Deviations from Hardy–Weinberg equilibrium: *P < 0.05, **P < 0.01.

The 39 primer pairs were also tested in *T. petaloideum* and *T. finetii*, and 26 of the developed primer pairs were found to be polymorphic (Table 3).

**Table 3:** Cross‐amplification and polymorphism analysis of 39 microsatellite markers developed for *Thalictrum smithii* in two related monoecious *Thalictrum* species.^a^^,^ b

Locus	*T. petaloideum*					*T. finetii*				
	Sample 1	Sample 2	Sample 3	Sample 4	Sample 5	Sample 1	Sample 2	Sample 3	Sample 4	Sample 5
Tsp04^c^	—	—	—	—	—	94/110	94/110	110	110/112/120	120
Tsp08^c^	—	—	161	161	161	—	—	—	—	—
Tsp09^c^	92/98	98	98	98	98	—	98	84/98	—	98
Tsp13^c^	—	—	—	—	—	—	—	169	—	—
Tsp15^c^	92/96	92/96	92/96	92/96	92/96	96	96	96	98	96
Tsp16^c^	—	—	—	—	—	102/106/116	102	102/106/116	102	—
Tsp17^c^	146	146	146/158	146	146/152/158	158	158	158	—	—
Tsp18^c^	225	213/217/225	203/213/217	203/213	213/217	215/217	213/217	213/215/219	—	219
Tsp19^c^	—	—	—	—	—	—	—	—	210	—
Tsp31^c^	—	202	202/204/208	202/204	—	202/204	202	204/208/212	212	212
Tsp32^c^	216	216	216/220	216	—	206	206	206	206	206
Tsp37^c^	101/103	99/101/103	97/99	97/101	97/99	95/99	99	95/99	101	107
Tsp39^c^	118/134/136	134/136/138	118/134/138	118/136	118/136	110	110	110	110	110
Tsp01	—	—	135	135/139	135/139	105/119	119/137	105/119	—	—
Tsp02	—	—	—	—	—	—	—	—	—	—
Tsp03	—	—	—	147	147	—	—	—	—	—
Tsp05	135/137/141	137/141	125	125/151	125/151	121/125/129	125/137	125	121/125/151	125/129/137
Tsp06	—	—	—	136	—	—	—	—	—	—
Tsp07	188	188	188	186	188	198	198/216	198/216	212	212
Tsp10	—	—	102/104	102	102/104	—	—	—	—	—
Tsp11	—	—	—	—	—	—	—	—	—	—
Tsp12	—	—	—	—	—	251	251	251	263	263
Tsp14	—	—	—	—	—	—	—	—	—	—
Tsp20	—	—	—	—	—	—	—	—	—	—
Tsp21	—	—	—	—	—	—	—	—	—	—
Tsp22	—	—	—	—	—	—	—	—	—	—
Tsp23	—	—	—	—	126	—	—	—	—	—
Tsp24	—	—	—	—	—	—	—	—	—	—
Tsp25	169	—	169	169	169	165/169	165/169	165	169/167	165
Tsp27	—	—	—	—	—	—	—	—	—	—
Tsp28	—	—	—	—	—	—	—	—	—	—
Tsp29	152/154	152/156	154/156	152	154/156	154	154	154	152/154	152/154
Tsp30	—	—	—	—	—	—	—	—	—	—
Tsp33	103	103	103	103	103	103/119	103/119	103/119	103/105	103/105
Tsp34	—	—	116	116	116	—	—	—	116	—
Tsp35	80/82	82	82	80	82	84	84	84	82	84
Tsp36	—	—	—	—	—	—	—	—	—	—
Tsp38	—	—	—	—	—	—	—	—	—	—
Tsp40	148/154	154	138/144	138/144	138/144	144	144	144	154	154

Note

— = no amplification product.

a See Appendix 1 for locality and voucher information.

b Values separated by a slash (/) represent band sizes, which vary as a result of either duplication(s) in other parts of the genome or nonspecific amplification.

c Loci marked with an asterisk have high levels of polymorphism and have been grouped at the beginning of the table to facilitate review of these loci and their cross‐amplification and polymorphism information.

## Conclusions

The SSR markers developed here can be used to estimate the levels of genetic diversity and gene flow in populations of *T. smithii* and to explore the evolutionary transitions in sexual systems in this genus from hermaphrodite to dioecy. Detailed information on the genetic consequences of sexual differentiation on *T. smithii* could also be obtained by using these SSR markers at larger scales.

## Data accessibility

Sequence information for the developed primers has been deposited to the National Center for Biotechnology Information (NCBI); GenBank accession numbers are provided in Table 1.

## Appendix 1 Location information for populations of *Thalictrum* used in the study and the sex ratios sampled. The voucher specimens are deposited in the Central South University Herbarium, Changsha, China.

**Table:**

Species	Population code	Population locality	Geographic coordinates	Sex ratio^a^	*N*	Voucher no.
*T. smithii* B. Boivin	SGC	Songgangcun, Sichuan Province, China	31.925939N, 102.108678E	0.87	F: 14, H: 20	CSUC605069
	XGLL	Xianggelilazhen, Sichuan Province, China	28.564108N, 100.351135E	0.59	F: 20, H: 20	CSUC607152
	YJX	Yajiangxian, Sichuan Province, China	30.033902N, 101.022107E	0.62	F: 20, H: 20	CSUC607145
*T. petaloideum* L.	LZP	Muli, Sichuan Province, China	28.026361N, 101.195139E	—	5	CSUC508033
*T. finetii* B. Boivin	KW	Muli, Sichuan Province, China	28.123889N, 101.213333E	—	5	CSUC508041

Note

F = female; H = hermaphrodite; N = number of individuals sampled.

a Sex ratio represents the frequency of hermaphrodites in each population.
